# Unusual mucoepidermoid carcinoma of the liver misdiagnosed as squamous cell carcinoma by intraoperative histological examination

**DOI:** 10.1186/1746-1596-9-24

**Published:** 2014-01-29

**Authors:** Xiao-qin Guo, Bin Li, Yang Li, Xiao-ying Tian, Zhi Li

**Affiliations:** 1 Department of Pathology, The First Affiliated Hospital, Sun Yat-sen University, 58, Zhongshan Road II, Guangzhou 510080, China; 2 School of Chinese Medicine, Hong Kong Baptist University, 7, Baptist University Road, Kowloon Tong, Hong Kong, China

**Keywords:** Mucoepidermoid carcinoma, Intrahepatic tumor, Histological features, Differential diagnosis

## Abstract

As rare condition, mucoepidermoid carcinoma may occur in liver although its etiology and pathogenesis is still unclear. We report here a case of intrahepatic mucoepidermoid carcinoma misdiagnosed as cholangiocarcinoma and squamous cell carcinoma by preoperative radiologic and intraoperative histological examinations, respectively. A 60-year-old woman presented with a 1-month history of progressive jaundice, epigastric discomfort, and weight loss with slightly increased carbohydrate antigen 19-9 (CA19-9). Computed tomography (CT) showed a large tumor, 8.0 cm in diameter, in the left lobe of the liver. A preliminary diagnosis of a cholangiocarcinoma of the liver was made. In the intraoperative histological examination, a diagnosis of squamous cell carcinoma was made based on predominantly invasive epidermoid cells with abundant keratinization and absence of mucin-producing cell component. However, postoperative histological diagnosis of the lesion was mucoepidermiod carcinoma of liver by thoroughly microscopical inspection and the presence of mucin-producing cells confirmed by Alcian blue staining. Despite surgical excision and chemotherapy, the tumor showed very aggressive malignancy with tumor recurrence. The patient died shortly afterward, surviving 6 months after surgery. Due to its rarity and distinct morphological features, mucoepidermoid carcinoma might be erroneously interpreted as squamous cell carcinoma by those who were not familiar with this condition in unusual locations. Therefore, removal of sufficient tissue from different portions of the lesion is essential for the surgeons and pathologists to make a precise diagnosis in the intraoperative histological examination.

**Virtual slide:**

The virtual slide(s) for this article can be found here: http://www.diagnosticpathology.diagnomx.eu/vs/4956311271136060

## Background

Mucoepidermoid carcinoma is a relatively common neoplasm of the salivary glands, which rarely arises in other sites, including esophagus, anal canal, skin of the breast, lachrymal sac, thymus, thyroid gland, lung or uterine cervix [1-4]. Primary intrahepatic mucoepidermoid carcinoma is rare tumor with only 17 cases described in the English literature so far [5-17]. Because of its relative rarity in liver, its etiology has not yet been elucidated. Terminal intrahepatic bile ducts or biliary congenital cysts have been proposed as a possible origin [5,7,8,11]. Histologically, mucoepidermoid carcinoma is characterized by squamoid (epidermoid), mucus producing and cells of intermediate type. The proportion of different cell types and their architectural configuration varies in and between tumors. In general, it is not difficult to distinguish mucoepidermoid carcinoma from other tumors arising from salivary glands by histopathological examination because of its distinct triphasic cellular morphology. However, mucoepidermoid carcinoma can be confused with squamous cell carcinoma when intermediate cells and epidermoid sometimes are prominent in the tumor. In particular, when the mucoepidermoid carcinoma occurs in an unusual site, a definite diagnosis might not be obtained without carefully histological examination. We present a case of mucoepidermoid carcinoma occurring in an old female patient that was misdiagnosed as cholangiocarcinoma and squamous cell carcinoma by both radiologic and intraoperative histopathological examination, respectively.

## Case presentation

**Figure 1:**
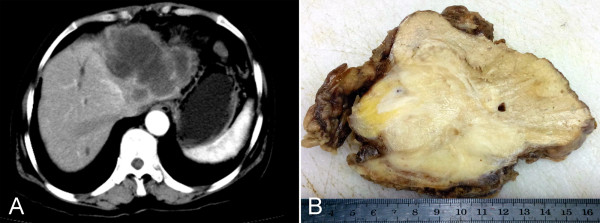
**Preoperative and gross findings of tumor. (A)** Computed tomography (CT) showed a large tumor lesion, 8.0 cm in diameter, in the left lobe of the liver. **(B)** Gross examination of resected liver mass showed an irregular, yellowish white solid tumor without a fibrous capsule. The border between the tumor and normal liver tissue was indistinct.

**Figure 2:**
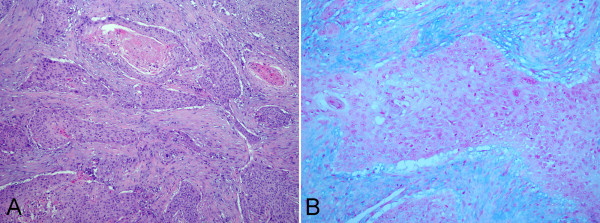
**Micrographs of liver mass in intraoperative histological examination. (A)** Intraoperative tumor tissue showed the tumor mass was predominantly composed of solid and invasive nests of epidermoid cells with abundant keratinization in desmoplastic stroma. **(B)** Alcian blue staining showed that there was no mucin-producing cells intermingled within the epidermoid cells nest. (**A**, HE staining with original magnification × 400; **B**, Alcian blue staining with original magnification × 400).

A 60-year-old Chinese woman, presented with a history of progressive jaundice, epigastric discomfort, anorexia, malaise, and weight loss for 1 month. She had no remarkable medical or family history and had not had viral hepatitis. Clinical examination revealed a deeply jaundiced, emaciated woman without ascites. A hard, irregular liver was palpable 4 cm below the xiphisternum. The results of relevant laboratory studies were as follows: total protein, 7.6 g/dl; albumin, 3.5 g/dl; total bilirubin, 513 u mol/L; GOT, 35U; and GPT, 23U. α-Fetoprotein (AFP) and carcinoembryonic antigen (CEA) were within the normal range and carbohydrate antigen 19-9 (CA19-9) was slightly increased to 50 mg/dl (normal range, 0-37 mg/dl). Hepatitis B viral antigen and hepatitis C antibody assays were negative. Abdominal ultrasound and abdominal computed tomography (CT) both showed a large liver tumor lesion, 8 cm in diameter involving the left lobe of the liver with intrahepatic bile duct stone (Figure 1). Therefore, a preliminary diagnosis of a cholangiocarcinoma of the liver was made, and the patient underwent left hepatic lobectomy with regional lymph node dissection. Intraoperative finding revealed an elastic hard tumor was found in the left lobe of the liver. The cut surface of the resected specimen showed an irregular, yellowish white solid tumor, measuring 8.5 × 6.5 × 3.0 cm, with central necrosis. The border between the tumor and normal liver tissue was indistinct (Figure 2A). A piece of tumor tissue was resected from the surface for intraoperative histological examination. Microscopically, the tumor was predominantly composed of nests of invasive epidermoid cells. Most of the tumor cells were epidermoid with intercellular bridges and keratinization. There were no distinct mucin-producing cells in the tissues (Figure 2B). Based on these findings, the diagnosis of squamous cell carcinoma of liver was made. The mass was totally removed.

## Pathological findings

**Figure 3:**
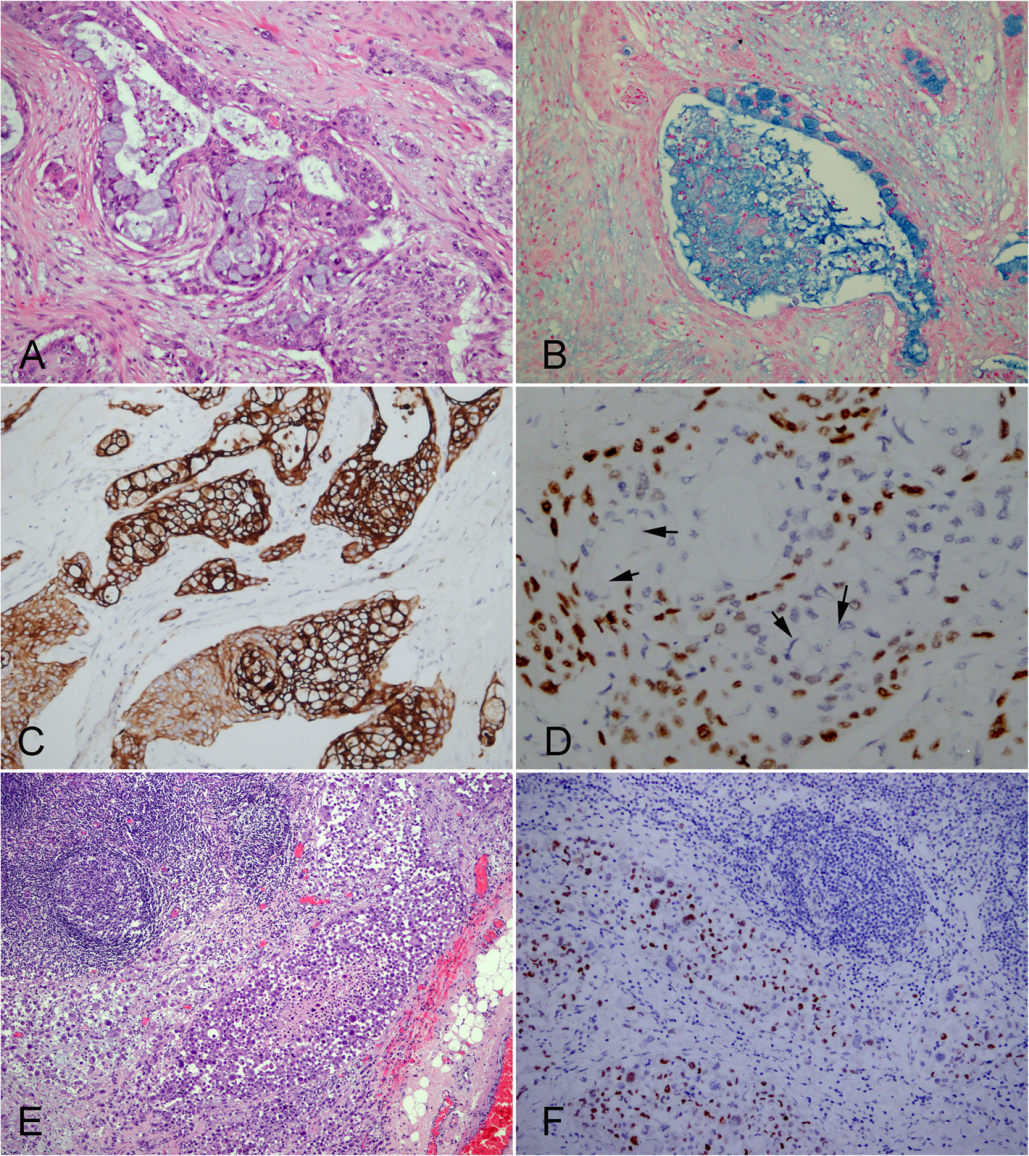
**Postoperative micrographs of liver mass. (A)** Postoperative histological examination of tumor exhibited that nests of malignant epidermoid cells were intimately mixed with mucus-producing cells. **(B)** The Alcian blue-positive material was seen in lumen of gland structure and the mucin-producing cells within the nest of epidermoid cells. **(C)** Tumor cells were diffusely positive for CK7. **(D)** The epidermoid cells were observed to be positive for p63, but the mucin-producing cells (black arrows) were p63-negative. **(E)** The lymph nodes metastasis of tumor was observed. **(F)** The metastatic tumor cells were also positive for p63 partially. (**A** and **E**, HE staining with original magnification × 400; **B**, Alcian blue staining with original magnification × 400; **C**, **D** and **F**, immunohistochemical staining with original magnification × 400).

After surgery, routine histological investigation was performed on the removed mass. Histopathological examination revealed that the main tumor was composed of solid and invasive nests of epidermoid cells with desmoplastic stroma. However, in some areas, not only squamous cells, but also mucin-producing and intermediate cells were observed in the tumor. These tumor cells were intermingled or intimately mixed with epidermoid cells, unlike adenosquamous carcinoma. Mucin-producing cells were cuboidal, columnar, or goblet-like. There was extensive necrosis accompanied by neural invasion, lymphatic and blood vessel invasion. Mitotic figures were frequent and there was remarkable cellular pleomorphism. There were several hepatic hilar lymph nodes metastasis found. Immunohistochemically, the epidermoid cells of the tumor were positive to pan-cytokeratin (CK), CK5/6 and p63, but mucin-producing cells were negative to CK5/6 and p63. Alcian blue staining revealed mucin in the cytoplasm of the mucin-producing cells. Based on these findings, the final diagnosis of tumor was revised as mucoepidermoid carcinoma (Figure 3). The histological grading of the tumor was evaluated according to the neural invasion, necrosis, mitotic rate and degree of maturation of cellular components [18]. The tumor was graded as high grade. The patient had an uneventful postoperative recovery. Since there was a possibility of tumor metastasis to another anatomical location, the patient was referred to a whole body positron emission tomography (PET)/CT study to search for the potentially secondary tumor, but no abnormality was found. After diagnosis, the patient received chemotherapy with gemcitabine, 5-fluorouracil and cisplatin. However, two months later she was admitted into another hospital with recurrent liver disease. She died shortly afterward, surviving 6 months after surgery. No postmortem was performed.

## Discussion

Mucoepidermoid carcinoma of liver is rare. In 1971, Pianzola and Drut reported the first case and suggested that this type of carcinoma arose from the terminal ramifications of bile canaliculi in association with squamous metaplasia [5]. However, the etiology and pathogenesis of mucoepidermoid carcinoma of the liver is still unclear. Some authors observed the remarkable neoplastic transformation of the normal duct lining epithelium, which suggested a possible of bile duct origination [16]. Some authors proposed that mucoepidermoid carcinoma of the liver might originate from a congenital cyst because the main tumors were located in the vicinity of multiple seromucinous cysts lined with columnar, cuboidal glandular epithelium with no connection to the biliary system and no bile content [8,11]. The electron micrographs revealed tonofilaments and confirmed the squamous nature of the tumor cells. Immunohistochemical analysis provided evidence of the ductal epithelial origin of this neoplasm with consistently positive for CK7 and negative for CK20. Therefore, the World Health Organization (WHO) accepted the designation of mucoepidermoid carcinoma of liver as a rare but distinct variant of intrahepatic cholangiocarcinoma [19]. It is speculated that the lining epithelia of the congenital cysts in the liver may be transformed into the pluripotential intermediate cells, which may differentiate into both mucus-secreting and squamous cells.

Despite its enigmatic histogenesis, the pathological diagnosis of mucoepidermoid carcinoma of liver is based on both mucin-producing and epidermoid malignant cells in intimately mixed nests [18]. But establishing a preoperative diagnosis is difficult because of the rarity of these tumors and the fact that there are no specific landmarks in the radiologic examinations. Conventional CT and ultrasonography usually show an intrahepatic mass and make the diagnosis of intrahepatic cholangiocarcinoma or hepatocellular carcinoma. Like the most of previously reported cases, the current case was diagnosed as intrahepatic cholangiocarcinoma in the preliminary radiologic examination. In intraoperative investigation, the tumor was predominantly composed of nests of invasive epidermoid cells in desmoplastic stroma without distinct mucin-producing cells component. Furthermore, most of the tumor cells were epidermoid with intercellular bridges and keratinization, which might be regarded as keratinizing pearl of tumor, gave the histological appearance of squamous cell carcinoma. Since the mucoepidermoid carcinoma is rare in liver, these morphological features might be erroneously interpreted squamous cell carcinoma by those who were not familiar with this condition. This might be the reason that led to the misdiagnosis of squamous cell carcinoma. In our case, it was difficult for pathologists to provide a precise diagnosis from a few very small tumor tissues in the intraoperative histological investigation. However, in routine histological examination, correct diagnosis was gained by thorough inspection and presence of mucin-producing cells component. Therefore, surgeons should be responsible for removing sufficient tumor tissue from different portions of the tumor even if the first specimen supports the radiologic diagnosis.

To our knowledge, only 17 cases of primary mucoepidermoid carcinoma of the liver have been described in the literature so far [5-17] (Table 1). The ratio of mucoepidermoid carcinoma of liver incidence in women and men is 8:9, which is different from this tumor occurring in salivary gland with a 3:2 female predilection. Intrahepatic mucoepidermoid carcinoma seems to occur frequently in elderly patients (ranged from 35 to 81 years with mean age of 60), although its counterpart in salivary gland occur most commonly in patients under 40 years old [18]. 11 of 17 cases had lymph nodes and (or) distant metastasis and 15 patients died of disease within 7 days to 11 months after excision, although several cases received chemotherapy after surgical resection, including our case. In laboratory data, more than half the cases showed high CEA or CA19-9 with normal AFP and was diagnosed preoperatively as cholangiocarcinoma or hepatocellular carcinoma. Only one case showed elevated the tumor marker of squamous cell carcinoma, suggesting a squamous component [15]. However, none of previously reported cases was diagnosed as mucoepidermoid carcinoma of the liver preoperatively. That indicates diagnosing primary mucoepidermoid carcinoma in the liver is clinically difficult because these tumors are rare and have no specific findings by radiological and laboratory examinations. Therefore, percutaneous biopsy is needed for this tumor to obtain a definite diagnosis preoperatively [17].

**Table 1:** Clinicopathological features of intrahepatic mucoepidermoid carcinoma described in present and previous reports

**No.**	**Authors (yr.)**	**Age (year)/Gender**	**Location/size (cm)**	**Clinical manifestation**	**Tumor markers**	**Preoperative diagnosis**	**Metastasis**	**Histological grading**	**Treatment**	**Outcome**
1	Pianzola LE [5]	44/M	RL/15.0	Abdominal pain	NA	Hydatid cyst	None	NA	Surgical excision	Liver failure and dead 45 days after surgery
2	Ho JC [6]	65/M	RL/8.0	Jaundice	NA	NA	Lymph node and pancreas	NA	Conservative	Complication and dead 14 days after biopsy
3		63/F	LL/6.0	Abdominal pain	NA	NA	Lymph node and pancreas	NA	Conservative	Dead 16 days after diagnosis
4	Koo J [7]	44/F	LL/12.0	Cholangitis and hepatomegaly	AFP < 5	CC	None	NA	Surgical excision + Chemotherapy	Recurrence and dead 6 months after surgery
5		66/M	CHD/4.0	Progressive jaundice	AFP < 5	NA	Lymph node	NA	Surgical excision	Died 1 week after surgery
6		62/M	CHD/1.5	Progressive jaundice	APF < 5	NA	None	NA	Surgical excision	Alive after10 months following-up
7	Katsuda S [8]	78/M	LL/11.0	Hepatomegaly	AFP = 12.5	HCC	Lymph node and lung, kidney	NA	Chemotherapy	Recurrence and dead 3 months
8	Kim YI [9]	35/M	LL/18.0	Abdominal pain	AFP < 5	NA	None	NA	Surgical excision	Alive after 1 year following-up
9	Lambrianides AL [10]	59/F	RL/18.0	Abdominal pain	NA	SCC	Kidney	NA	Conservative	Dead 14 days after diagnosis
10	Hayashi I [11]	46/F	LL/3.0	Abdominal pain	AFP = 20	NA	None	High-grade	Surgical excision	Recurrence and dead 11 months after surgery
11	Di Palma S [12]	66/F	LL/9.5	Abdominal pain	CA19-9 = 500, CEA < 2	NA	Diaphragm and pericardial	High-grade	Surgical excision	Whole body metastasis and dead 6 months after surgery
12	Kim JM [13]	68/M	LL/10.0	NA	AFP < 5	NA	None	NA	Conservative	NA
13	Shuangshoti S Jr [14]	64/M	LL/5.0	Jaundice	NA	NA	Lymph node	High-grade	Conservative	Intestinal bleeding and dead 7 days after diagnosis
14	Kang H [15]	52/M	LL/7.0	Epigastric pain	AFP < 5, SCC = 14.1	HCC	Lymph node	High-grade	Surgical excision	Dead 6 month after surgery
15	Choi D [16]	69/F	RL/16.0	Abdominal pain	CA19-9 = 240	Liver abscess	Diaphragm	NA	Surgical excision	Recurrence and dead 4 months after surgery
16	Arakawa Y [17]	81/F	RL/10.0	Fever	CA19-9 = 14893	CC	Lymph node	High-grade	Chemotherapy	Cholangitis and dead 4 months after diagnosis
17	The present case	60/F	LL/8.5	Jaundice and epigastric pain	CA19-9 = 50	CC	Lymph node	High-grade	Surgical excision + chemotherapy	Recurrence and dead 6 months after surgery

M, male; F, female; RL, right lobe of liver; LL, left lobe of liver; CHD, common hepatic duct; AFP, a-fetoprotein; CA19-9, carbohydrate antigen 19–9; CEA, carcinoembryonic antigen; CC, cholangiocarcinoma; HCC, hepatocellular carcinoma; SCC, squamous cell carcinoma; NA, not available.

Mucoepidermoid carcinomas arising in salivary gland and central airway of lung should be distinguished from other salivary gland-type tumors, such as adenoid cystic carcinoma, clear cell carcinoma, epithelial-myoepithelial carcinoma, and pleomorphic adenoma, especially in pediatric population [20,21]. Recent study has demonstrated that decreased expression of maspin (mammary serine protease inhibitor) and marked increase of MCM2 (minichromosome maintance-2) expression support the diagnosis of high-grade mucoepidermoid carcinoma [22]. However, due to their rarity and distinct morphological features, primary mucoepidermoid carcinoma of the liver is sometimes misdiagnosed as cholangiocarcinoma with squamous metaplasia, adenosquamous carcinoma or squamous cell carcinoma because the proportion of different cell types and their architectural configuration varies in and between tumors. Higuchi et al. emphasized the importance of differential diagnosis for adenosquamous carcinoma, adenoacanthoma, and mucoepidermoid carcinoma when they occurred in liver [23]. In the present case, the epidermoid cell population and keratinization were prominent in the tumor, which might be erroneously interpreted as squamous cell carcinoma. However, mucin-producing cell is absent in the squamous cell carcinoma, which can be demonstrated in majority of tumor by Alcian blue and diastase-PAS staining. Sufficient tissue from different parts of the tumor and thorough inspection to find the mucin-producing cells will facilitate the precise diagnosis of mucoepidermoid carcinoma. Adenosquamous carcinoma combines an adenocarcinoma and squamous cell carcinoma. Unlike mucoepidermoid carcinoma, the two components of adenosquamous carcinoma show either as separate areas within the tumor or admixed. However, mucin-secreting cells were observed to intermingle or intimately mixed with epidermoid cells in mucoepidermoid carcinoma. In rare condition, cholangiocarcinomas containing discrete foci of benign-appearing squamous metaplasia are termed adenocarcinoma with squamous differentiation or adenoacanthoma, which might be confused with mucoepidermoid carcinoma or adenosquamous carcinoma. But the absence of mucin-secreting cells within the foci of squamous metaplasia will help to distinguish this tumor from with mucoepidermoid carcinoma.

Mucoepidermoid carcinoma of the liver is regarded as an aggressive tumor with poor prognosis despite surgical treatment. Recent study suggested that aberrant expression of p53 and mdm-2 correlated with the high histological grade of the tumor and were associated with tumor behavior and local recurrence [24]. Among those reported cases, more than 10 patients died within 6 months after the initial diagnosis [5-8,10,12,14-17]. Our patient survived for only 6 months after aggressive surgical intervention and chemotherapy. The specific chemotherapy regimen for mucoepidermoid carcinoma of the liver has not yet been established. The reported chemotherapy regimens for intrahepatic mucoepidermoid carcinoma were based on the standards for it in salivary glands, including adriamycin/mitomycin combination, methotrexate/fluorouracil combination, or ormitomycin as a single agent. Some researchers suggested that molecular targeted chemotherapy including an anti-HER2 or anti-epidermal growth factor (EGFR)-based regimen might be the most promising strategy for treatment of salivary gland cancers [25-27]. However, the overexpression or gene amplification of HER2 in intrahepatic mucoepidermoid carcinoma has not been clarified yet, although it could be detected in up to one-third of patients with mucoepidermoid carcinoma in the salivary glands. Therefore, researchers suggest multi-institutional studies are needed to clarify the histogenesis and biological behavior of primary mucoepidermoid carcinoma of the liver [16].

## Conclusion

In conclusion, we reported an unusual case of intrahepatic mucoepidermoid carcinoma misdiagnosed as squamous cell carcinoma by intraoperative histological examination. Due to its rarity, mucoepidermoid carcinoma may be erroneously interpreted as squamous cell carcinoma when the epidermoid cell population and keratinization were prominent in the tumor. Therefore, more precise diagnosis for those rare cases in the intraoperative histological examination is facilitated by obtaining sufficient tissue from different parts of the lesion.

## Consent

Written informed consent was obtained from the patient's family for publication of this case report and any accompanying images. A copy of the written consent is available for review by the Editor-in-Chief of this journal.

## Competing interests

The authors declare that we have no competing interests.

## Authors’ contributions

XQG and BL made contributions to acquisition of clinical data, and analysis of the histological features by H&E staining and immunoassays. They are joint first co-authors and made an equal contribution to this work. YL carries on the immunohistochemical and special staining. XYT drafted the manuscript. ZL revised manuscript critically for important intellectual content and had given final approval of the version to be published. All authors read and approved the final manuscript.
